# miR-372 down-regulates the oncogene ATAD2 to influence hepatocellular carcinoma proliferation and metastasis

**DOI:** 10.1186/1471-2407-14-107

**Published:** 2014-02-19

**Authors:** Gang Wu, Haiyang Liu, Hui He, Yawei Wang, Xiaojun Lu, Yanqiu Yu, Shuguan Xia, Xiangyu Meng, Yongfeng Liu

**Affiliations:** 1 Department of General Surgery, the First Affiliated Hospital of China Medical University, Shenyang, Liaoning 110001, China; 2 Department of Pathophysiology, China Medical University, Shenyang, Liaoning 110001, China

## Abstract

**Background:**

ATAD2 is associated with many cellular processes, such as cell growth, migration and invasion. However, no studies have been conducted on the molecular biological function of the ATAD2 gene in hepatocellular carcinoma (HCC).

**Methods:**

The protein and mRNA level expression of ATAD2 was examined in tissues and cell lines. Prognostic significance was analyzed by the Kaplan-Meier survival method and Cox regression. ATAD2 knockdown was used to analyze cell proliferation and invasion. The upstream and downstream of ATAD2 was analyzed by RT^2^ Profiler™ PCR array and luciferasex fluorescence system.

**Results:**

ATAD2 was highly expressed in liver cancer samples and correlated with poor survival. High ATAD2 expression was positively correlated with metastasis (P = 0.005) and was an independent prognostic factor in HCC (P = 0.001). ATAD2 depletion by RNA interference reduced their capacity for invasion and proliferation and led to a G1 phase arrest in vitro. Further study revealed that miR-372 was an upstream target of ATAD2 as miR-372 was bound directly to its 3′ untranslated region (3′ UTR). In addition, ATAD2 knockdown was found to extremely up-regulate APC expression and down-regulate CTNNA1 at the mRNA level.

**Conclusions:**

The findings demonstrated that miR-372 suppressed the expression of ATAD2, which was highly expressed in HCC and exerted a proto-oncogene effect in hepatic carcinogenesis. In conclusion, ATAD2 may promote HCC progression.

## Background

HCC is the fifth most common malignant tumor worldwide, with an incidence of approximately 626,000 cases each year [1,2]. In China and Southeast Asia, HCC is highly associated with viral hepatitis B and cirrhosis [3]. The prognosis of patients with HCC has largely improved due to extensive advances in surgical techniques and diagnostic methods in recent years. However, the long-term prognosis is still unsatisfactory, largely due to the high recurrence and invasion rates even after resection (50-70% at five years) [4,5]. Unfortunately, little is known with respect to the molecular mechanisms underlying this aggressive behavior. Therefore, there is great demand to find a reliable prognostic biomarker, which may help clinicians predict the characteristics of the malignancy and decrease the rate of unfavorable outcomes in a high-risk population.

ATAD2 (ATPase family AAA domain-containing protein 2), a member of the AAA + ATPase family of proteins, was identified by microarray analysis [6], contains both a bromodomain and an ATPase domain, and maps to chromosome 8q24 in a region that is frequently amplified in cancer [7]. The structure of ATAD2 suggests that it has functions related to genome regulation, including cell proliferation, differentiation and apoptosis. Studies have revealed that ATAD2 is highly expressed in several types of tumors such as breast cancer, lung cancer, and large B-cell lymphoma [8-13]. And recently Huang Q et al. has reported that a novel, highly up-regulated exon-exon junction was detected in ATAD2 gene by RNA-seq and the gene was highly expressed in HCC tissues [14]. However, there have been no studies on the gene function and prognosis associated with ATAD2 in HCC.

In the present study, we detected the expression of ATAD2 in HCC and matched adjacent non-cancerous liver tissues. Different methods were used to determine the relationships between the expression of ATAD2, its clinical relevance, and the overall survival (OS) after resection. In addition, the effects of ATAD2 expression on cell invasion and metastasis were investigated in SMMC7721, QGY-7701, Bel-7402, PLC5, Huh7, HCCLM3, HepG2, and LO2 cell lines using small interfering RNAs. Also the miR-372 was identified as a direct and functional target for ATAD2 in hepatic carcinogenesis. Therefore, both ATAD2 and miR-372 appear to be good targets to study in our research.

## Methods

### Patient tissue samples

Primary tumor samples were obtained from 129 patients (106 males and 23 females) who were diagnosed with HCC and had undergone routine hepatic resection in the First Affiliated Hospital of China Medical University between January 2006 and December 2009. The follow-up period for survivors was 5 years. None of the patients had received preoperative radiotherapy or chemotherapy prior to surgical resection. The histological diagnosis and differentiation were evaluated independently by two pathologists according to the WHO classification system [15]. The clinicopathological features are shown in Table 1. Fresh specimens were snap-frozen in liquid nitrogen and stored at -70°C immediately after resection for the extraction of RNA and protein. The project protocol was approved by the Institutional Ethics Committee of China Medical University prior to the initiation of the study. All patients provided written informed consent for the use of the tumor tissues for clinical research.

**Table 1:** Distribution of ATAD2 and Clinicopathological Characteristics in HCC patients

**Characteristics**	**Number of patients**	**ATAD2 overexpression**	**Negative or weak expression**	**P**
Total cases	129	83	46	
Age (years)				
≥60	54	34(63.0%)	20(37.0%)	0.782
<60	75	49(65.3%)	26(34.7%)	
Gender				
Male	106	69(65.1%)	37(34.9%)	0.701
Female	23	14(60.9%)	9(39.1%)	
Tumor size				
≥ 5cm	83	53(63.9%)	30(36.1%)	0.877
< 5cm	46	30(65.2%)	16(34.8%)	
Metastasis				
Yes	72	54(75%)	18(25%)	0.005
No	57	29(50.9%)	28(49.1%)	
HBsAg status				
Positive	73	43(58.9%)	30(41.1%)	0.141
Negative	56	40(71.4%)	16(28.6%)	
Tumor differentiation				
WD	54	34(63.0%)	20(37.0%)	0.955
MD	60	39(65.0%)	21(35.0%)	
PD	15	10(66.7%)	5(33.3%)	
Cirrhosis				
Yes	86	58(67.4%)	28(32.6%)	0.298
No	43	25(58.1%)	18(41.9%)	
Serum AFP				
<200 ng/dl	41	29(70.7%)	12(29.3%)	0.301
≥200 ng/dl	88	54(61.4%)	34(38.6%)	
Tumor stage				
TNM I+II	42	25(59.5%)	17(40.5%)	0.427
TNM III+IV	87	58(66.7%)	29(33.3%)	

### Liver cancer cell lines and cell cultures

The liver cancer cell lines, SMMC7721, QGY-7701, Bel-7402, PLC5, Huh7, HCCLM3, HepG2, and the normal liver cell line, LO2, were obtained from the Shanghai Cell Bank (Shanghai, China). SMMC7721, QGY-7701, Bel-7402 and PLC5 cells were cultured in RPMI-1640 (Invitrogen, Carlsbad, CA). Huh7, HCCLM3, HepG2 and LO2 cells were grown in DMEM (Invitrogen). All media were supplemented with 10% fetal calf serum (Invitrogen) and 100 IU/ml penicillin (Sigma, St. Louis, MO).

### RNA preparation and quantitative real-time PCR

Total RNA was extracted according to the manufacturer’s instructions. RT-qPCR was performed using a SYBR Premix Ex Taq (TaKaRa) on a Thermal Cycler Dice Real Time System (TaKaRa) with the following protocol: 30 s at 95°C followed by two-step PCR for 40 cycles of 95°C for 5 s and 64°C for 30 s. Each reaction was performed as we previously described [3]. The primer sequences were as follows: ATAD2 forward, 5′-GGAATCCCAAACCACTGGACA-3′;

ATAD2 reverse, 5′-GGTAGCGTCGTCGTAAAGCACA-3′;

GAPDH forward, 5′-ATAGCACAGCCTGGATAGCAACGTAC-3′;

GAPDH reverse, 5′-CACCTTCTACAATGAGCTGCGTGTG-3′.

### Western blots

Total protein was extracted from tumor tissues, non-tumor adjacent tissues and liver cancer cell lines using the Nuclear and Cytoplasmic Protein Extraction Kit (Beyotime, China). Fifty micrograms of total nuclear protein was separated by SDS-PAGE and then electrotransferred to PVDF membranes (Millipore, Billerica, MA, USA). Milk (5%) was used to block membranes for 2 hours at room temperature. After blocking, primary antibodies, including ATAD2, APC, CTNNA1 rabbit polyclonal antibody and GAPDH mouse polyclonal antibody (both diluted at 1:1000, Sigma, St. Louis, MO, USA), were incubated with the membranes overnight at 4°C. The secondary antibodies were then incubated for 2 hours at room temperature. Protein bands were identified using an ECL system (Millipore, Bedford, MA, USA)

### Immunohistochemistry

ATAD2 expression was analyzed on paraffin-embedded specimens from 129 patients. Tissue sections of 4-μm thickness were deparaffinized in xylene and dehydrated before antigen retrieval for 5 min with an autoclave. Hydrogen peroxide (0.3%) was used to block endogenous peroxidase activity; nonspecific immunoglobulin binding sites were blocked by normal goat serum for 30 min at 37°C. Tissue sections were incubated with anti-ATAD2 (1:200, Sigma) overnight at 4°C. Biotinylated goat anti-rabbit immunoglobulin G was used as a secondary antibody (1:300, Sigma). After washing, the sections were incubated with streptavidin-biotin conjugated with HRP for 30 min, and the peroxidase reaction was developed with 3, 3′-diaminobenzidine tetrahydrochloride (DAB).

### Semi-quantitative assessment and scoring

ATAD2 expression levels were scored semiquantitatively according to the percent of positively stained cells combined with the staining intensity. Samples were considered positive for ATAD2 if the nucleus or cytoplasm of the sample cells presented a positive staining. The percent positivity was defined as “0” if 0%, “1” if 1-10%, “2” if 11-50%, “3” if 51-80%, and “4” if >80%. The staining intensity was scored as “0” (no staining), “1” (weakly stained), “2” (moderately stained) and “3” (strongly stained). Both the percent positivity and the staining intensity were assessed by two doubly blinded investigators. The ATAD2 expression score was calculated from the value of percent positivity score × the staining intensity score. This value thus ranged from 0 to 12, and the tumors were classified into the following: negative (-), score 0; lower expression (1+), score 1–4; moderate expression (2+), score 5–8; and strong expression (3+), score 9–12. The immunohistochemical ATAD2 staining was grouped into two categories: low expression (0/1+) and high expression (2+/3+).

### Depletion of ATAD2 by small interfering RNAs

On-TargetPlus SMARTpool siRNA for ATAD2 (No. LU-017603-00-0002) and On-TargetPlus siControl (D-001810-01-20) were purchased from Dharmacon. For transfections, 24 h after the cells were seeded in a 6-well plate, they were transfected with ATAD2 siRNA or control siRNA using DharmaFECT 1 according to the manufacturer’s protocol. The mRNA and protein levels were detected 48 h later.

### Cell cycle analysis

Huh7 and HCCLM3 cells in 6-well plates were transfected with ATAD2 siRNA or negative control siRNA. After 48 h of transfection, cells seeded at a density of 5 × 10^5^ per well were trypsinized, fixed with 70% ethanol at 4°C, and washed with PBS. A quantity of 100 μL RNase A was added, and the mixture was incubated in a 37°C water bath for 30 min. An additional 400 μL PI staining solution was added and incubated at 4°C in the dark for 30 min; a computer was then used to detect and record the red fluorescence upon excitation at a wavelength of 488 nm.

### CCK8 and Colony formation assay

Cells were plated in 96-well plates in media containing 10% FBS at approximately 2,000 cells per well, 24 h after transfection. Then, 10 μl of CCK8 (Thiazolyl Blue) solution was added to each well and incubated for 1 h at 37°C. The results were quantified spectrophotometrically using a test wavelength of 450 nm.

After transfection, logarithmic growth phase cells in monolayer culture were prepared for the colony formation assay. Cells were plated in 6-well plates in media containing 10% FBS at approximately 200 cells per well. Colony formation was then allowed to proceed for two weeks. Cells were washed with 1 ml of PBS, fixed, stained with 500 μl of 0.1% crystal violet solution for 20 min, and finally washed three times with 1 ml of water. The fixed cell colonies were allowed to air dry. The clone formation rate was calculated.

### Cell invasion and migration assay

Huh7 and HCCLM3 cells were infected with ATAD2 siRNA for 48 h. Cells were then seeded onto a synthetic basement membrane present in the inset of a 24-well culture plate. In the invasion assay, polycarbonate filters coated with 50 μL Matrigel (1:9, BD Bioscience) were placed in a Transwell chamber (Costar). In the migration assay, no Matrigel was placed in the chambers. Fetal bovine serum was added to the lower chamber as a chemoattractant. Cells were then incubated at 37°C and allowed to invade through the Matrigel barrier for hours. After incubation, filters were fixed and stained with 0.1% crystal violet solution. Non-invading cells were removed using a cotton swab, and invading cells on the underside of the filter were counted with an inverted microscope.

### Real-time polymerase chain reaction gene array

Forty-eight hours after siRNA knockdown, RNA was extracted from the cells using Trizol (Invitrogen) and cleaned with the RNeasy_MinEluteTM Cleanup Kit (Qiagen, Valencia, CA). Subsequently, total RNA was reverse transcribed using SuperScript III Reverse Transcriptase (Qiagen), and complementary DNA was amplified by the polymerase chain reaction (PCR) using 2_Super Array PCR master mix (Qiagen). Real-time PCR was then performed on each sample with the Human Tumor Metastasis RT2 ProfilerTM PCR Array (SuperArray Bioscience) in a Thermal Cycler Dice Real Time System (Takara TP800, Japan) according to the manufacturer’s instructions. Data were normalized to GAPDH levels by the 2^-ΔΔCt^ method.

### Luciferase assays

293 T cells were plated into 24-well plates at 80% confluence 24 hours before transfection. A mixture of 200 ng pGL3-3′ UTR, 700 ng pGV214-miR-372, and 100 ng Renilla luciferase plasmid were transfected into 293 T cells using Lipofectamine 2000. Firefly and Renilla luciferase activities were calculated using a dual-luciferase reporter system (Promega, Madison, WI).

### Statistical analysis

SPSS 17.0 software for Windows was used. The association between ATAD2 expression and HCC patients’ clinicopathological features was evaluated by the χ2 test. The Wilcoxon test was performed to compare data from the densitometry analysis of mRNA and protein expression. The Kaplan-Meier method was used to calculate the patients’ survival by a log-rank test. A Cox repression model was performed for the univariate and multivariate analysis of prognostic variables. All P values reported are two-sided, and P < 0.05 is considered statistically significant.

## Results

### Expression and Clinical significance of ATAD2 protein expression in HCC

**Figure 1:**
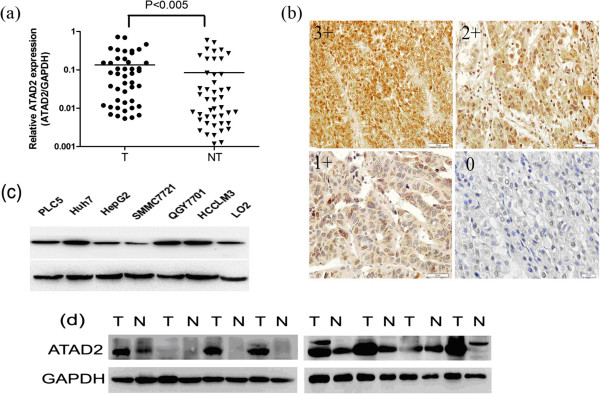
**The ATAD2 expression level was higher in HCC samples and liver cancer cell lines. (a)** The ATAD2 mRNA expression was up-regulated in tumors compared to the paired nontumour patient samples. **(b)** IHC staining of ATAD2 in HCC. **(c)** Huh7 and HCCLM3 cells showed relatively higher levels of ATAD2 expression than other liver cancer cell lines. **(d)** ATAD2 protein expression in each of the tumor and paired non-tumor samples was determined by Western blotting.

The median ATAD2 expression level in primary HCCs was nearly two-fold higher than in non-tumorous livers (median expression = 0.158 and 0.083, respectively), (P < 0.05 [Wilcoxon signed-rank test]) (Figure 1a). ATAD2 was overexpressed in 83 of 129 tumor samples (83/129, 64.3%) according to IHC. The ATAD2 protein appeared to be expressed in both the nucleus and the cytoplasm of tumor cells, with stronger expression in the nucleus (Figure 1b). Compared to the normal cell line LO2, Huh7 and HCCLM3 cells showed relatively higher levels of ATAD2 expression in seven liver cancer cell lines by Western blotting (Figure 1c). At the protein level, 6/8 (75%) HCC samples showed upregulated ATAD2 expression compared to their adjacent normal liver tissues (Figure 1d).

### Association of ATAD2 expression with HCC patient clinical outcome

**Figure 2:**
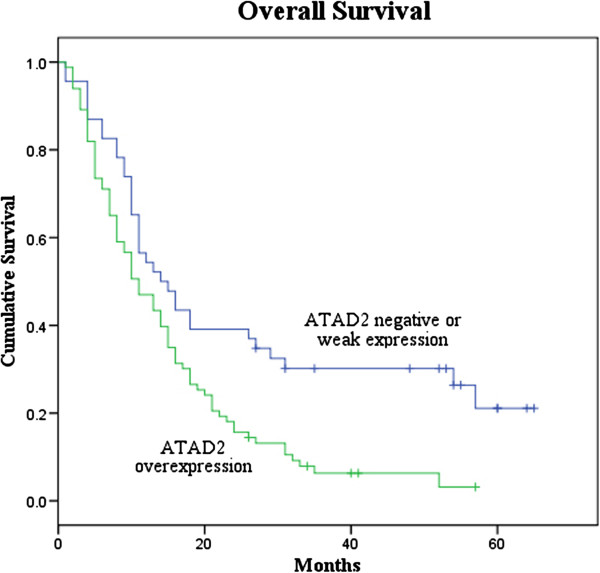
**Overall survival of HCC patients in relation to ATAD2 status.** Survival of HCC patients with low ATAD2 expression versus high expression.

Patients with high ATAD2 expression had metastases and poorer prognosis (Table 1). The overall survival was significantly lower in patients with high ATAD2 expression than in patients with low ATAD2 expression (P < 0.05, Figure 2). In addition, a multivariate analysis demonstrated that ATAD2 status, the tumor size, metastasis and the AFP status were significant prognostic factors for HCC patients (Table 2).

**Table 2:** Univariate and multivariate analyses of individual parameters for correlations with overall survival rate: Cox proportional hazards model

**Variables**	**Univariate**			**Multivariate**		
	**HR**	**CI (95%)**	**P value**	**HR**	**CI (95%)**	**P value**
ATAD2	1.843	1.220-2.784	0.004^a^	2.221	1.444-3.416	0.001^a^
Age	1.253	0.856-1.542	0.407			
Gender	1.176	0.601-1.583	0.921			
Tumor stage	1.122	0.920-1.367	0.255			
HBsAg	1.150	0.792-1.670	0.464			
Tumor differentiation	1.028	0.784-1.347	0.843			
Metastasis	1.816	1.232-2.678	0.003^a^	1.822	1.221-2.718	0.003^a^
Tumor size	1.573	1.059-2.336	0.025^a^	1.909	1.276-2.855	0.002^a^
Serum AFP	1.670	1.110-2.512	0.014^a^	1.992	1.306-3.038	0.001^a^
Liver cirrhosis	1.056	0.640-1.429	0.827			

*Abbreviation:* HR, Hazard Ratio; CI, Confidence Interval.

^a.^Statistically significant (p < 0.05).

### Depletion of ATAD2 inhibits tumor cell growth in liver cancer cell lines

**Figure 3:**
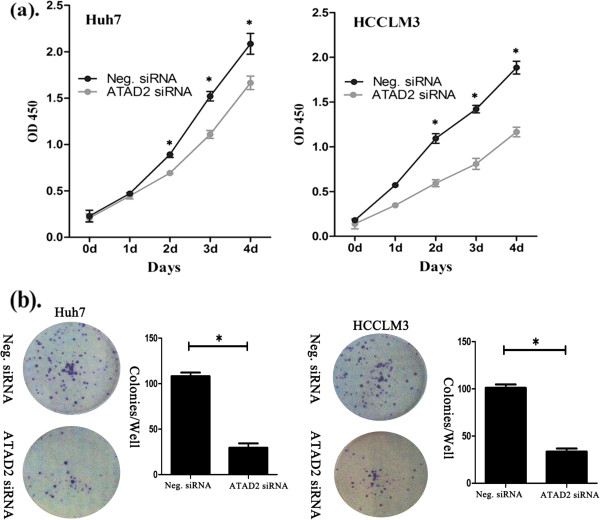
**The CCK8 assay was performed after ATAD2 siRNA treatment. (a)** A reduction of absorbance was observed (P < 0.05). **(b)** Clonogenic assays were performed with ATAD2-depleted cancer cells. The number of colonies formed by cells treated with ATAD2 siRNA was far fewer than that of control siRNA-treated cells (P < 0.05).

**Figure 4:**
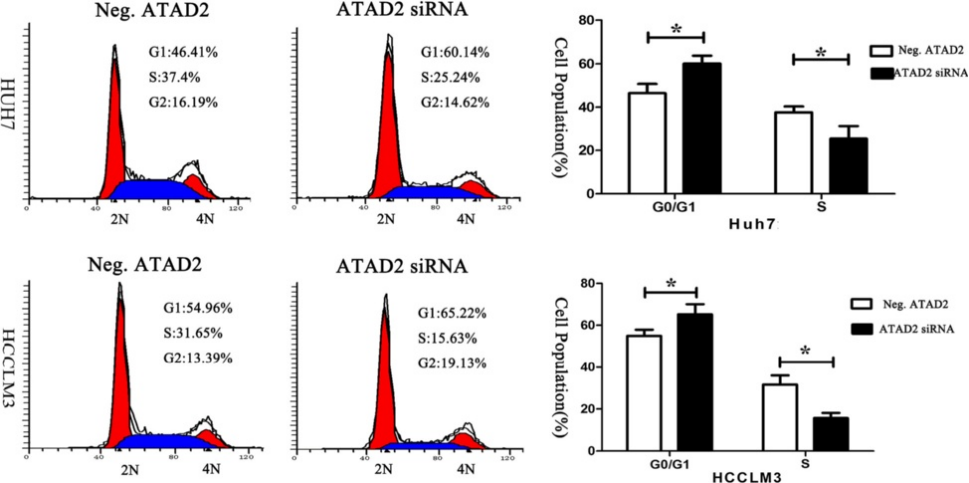
siRNA-mediated ATAD2 knockdown reduced Huh7 and HCCLM3 cell proliferation and led to a G1 phase cell cycle arrest.

A significant reduction in the proliferation rate was observed with the CCK8 assay 2 days after transfection with ATAD2 siRNA when compared to negative control siRNA (Figure 3a). Consistent with the CCK8 assay, the depletion of ATAD2 in Huh7 (Neg. siRNA vs. ATAD2 siRNA: 124 ± 16 vs. 36 ± 10, P < 0.001) and HCCLM3 (Neg. siRNA vs. ATAD2 siRNA: 102 ± 22 vs. 39 ± 13, P < 0.001) cells led to a significant reduction in the number and size of foci (Figure 3b). The DNA content determined using flow cytometry demonstrated that ATAD2 siRNA transfection increased the percentage of cells in G1 phase and decreased those in the S phase in both cell lines (Figure 4).

### Invasive and migratory capacity of liver cancer cells is decreased by ATAD2 knockdown

**Figure 5:**
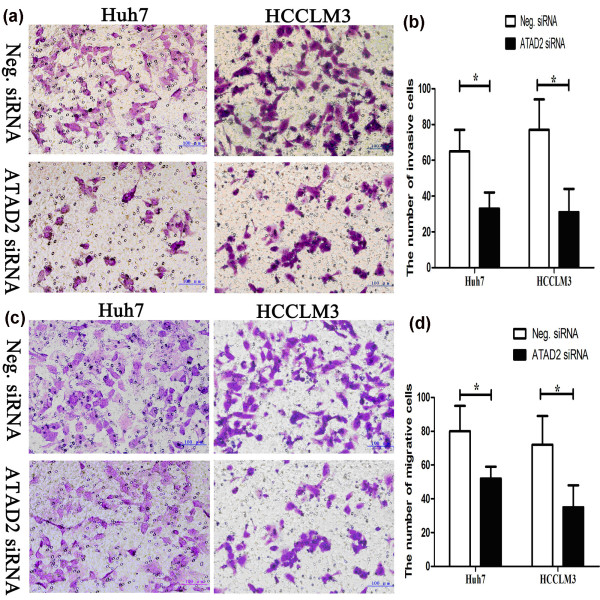
**Transwell assays of Huh7 and HCCLM3 cells transfected with control and ATAD2 siRNA. (a)**. ATAD2 depletion had a measurable inhibitory effect on cell invasion in both cell lines. **(b)**. The numbers of invading cells were counted, and a significant difference was observed (*P < 0.05). **(c)**. A significant difference was observed in both cell lines and **(d)** the numbers of migrating cells were counted (*P < 0.05).

Cell invasion and migration assays demonstrated that the Huh7 and HCCLM3 liver cancer cell lines that were transfected with ATAD2 siRNA displayed more attenuated invasive and migratory capacities than those of the negative controls (Figure 5).

The depletion of ATAD2 in Huh7 (control vs. ATAD2 siRNA: 63 ± 11 vs. 33 ± 7, *P < 0.05) and HCCLM3 (control vs. ATAD2 siRNA: 78 ± 14 vs. 32 ± 9, *P < 0.05) cells led to a significant reduction in invasive cells (Figure 5a-b). The migration of Huh7 (control vs. ATAD2 siRNA: 80 ± 11 vs. 51 ± 6,*P < 0.05) and HCCLM3 (control vs. ATAD2 siRNA: 70 ± 12 vs. 31 ± 8, *P < 0.05) cells was also significantly reduced (Figure 5c-d).

### ATAD2 regulated APC and CTNNA1 expression in HCC cells

**Figure 6:**
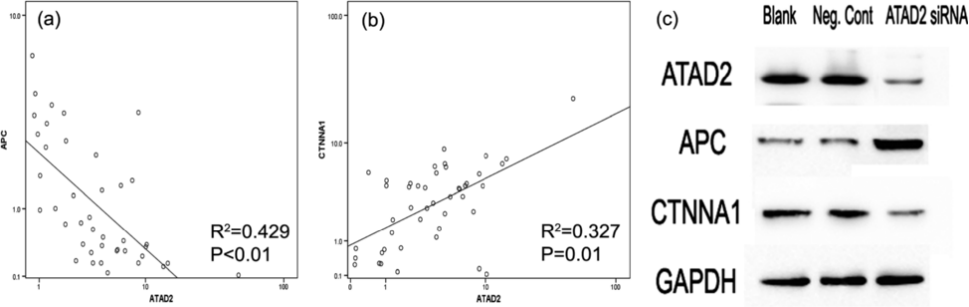
**APC and CTNNA1 expression was associated with ATAD2 in 45 HCC tissues. (a)** Real-time PCR results showed ATAD2 and APC expression was negatively correlated in HCC tissues (P < 0.01). **(b)** ATAD2 expression was positively correlated with CTNNA1 (P < 0.01). **(c)** At the protein level, the same trend was observed by Western blotting.

To obtain further insight into the functions of ATAD2 in HCC cell invasion and metastasis, the mRNA expression profile of siATAD2-transfected Huh7 cells was compared with that of si-negative transfected cells using a Human Tumor Metastasis RT^2^ Profiler™ PCR Array containing 84 cell metastasis-related genes. Six upregulated genes and five downregulated genes (at least 2-fold) were identified in siATAD2-transfected Huh7 cells compared to si-negative transfected controls (Additional file 1: Table S1). Subsequently, APC and CTNNA1, which showed the greatest fold change (3-fold or more) after ATAD2 knockdown, were selected and analyzed with ATAD2 in 45 HCC tissues by real time PCR using linear correlation statistics (Figure 6a and b). Consistent with the results from the real-time PCR array, the expression levels of ATAD2 and APC mRNA were negatively correlated (R^2^ = 0.429, P < 0.01), and ATAD2 expression was positively correlated with CTNNA1 in HCC tissues (R^2^ = 0.327, P = 0.01). At the protein level, the same trend was observed by Western blotting (Figure 6c).

### Oncogenic ATAD2 was a direct downstream target for miR-372

**Figure 7:**
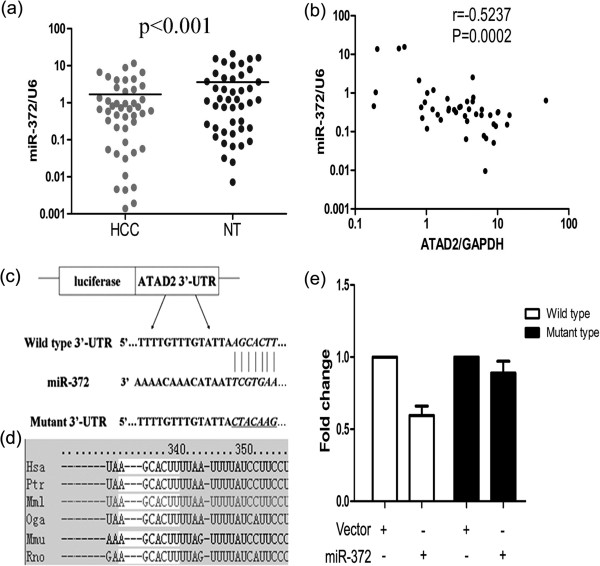
**The relationship between miR-372 and ATAD2. (a)** Significant down-regulation of miR-372 in paired HCC/NT samples was defined as a log_2_-fold change < -1. **(b)** miR-372 expression was negatively correlated with ATAD2 expression in 45 paired samples. **(c)** Luciferase assay with wild-type and miR-372 binding site-mutated ATAD2-3′ UTR in the presence of a miR-372 expression plasmid. Mutations are underlined and italicized. **(d)** miR-372 binding site in the ATAD2 3′ UTR in different species. The seed sequence is under the white box. **(e)** Relative luciferase activity analyses. The relative luciferase activity of the pGV214 group was set as 1.

We found that the miR-372 expression level was lower in HCC than in adjacent normal liver tissues (Figure 7a) and that it was negatively correlated with ATAD2 expression (r = -0.5237, P = 0.0002; Figure 7b) by RT-qPCR. According to prediction websites (TargetScan, PicTar and miRanda), we searched for the target genes of miR-372 and found that ATAD2 contains one potential miR-372 binding site in its 3′ UTR (Figure 7c). This sequence is highly conserved across different species (human, chimpanzee, mouse, rat, pig, and rabbit) (Figure 7d). Therefore, we constructed vectors containing the wild-type or mutant 3′ UTR of ATAD2 fused downstream of the firefly luciferase gene. The wild-type or mutant vector was co-transfected into 293 T cells with a miR-372 expression construct, and the transfection efficiency was normalized by co-transfection with a Renilla luciferase vector. As shown in Figure 7e, miR-372 significantly decreased the relative luciferase activity of the wild-type ATAD2 3′ UTR (40%) compared to the mutant 3′ UTR, indicating that miR-372 could directly bind to the 3′ UTR of ATAD2. Thus, we suggest that ATAD2 is a direct target of miR-372.

## Discussion

It was demonstrated that ATAD2 is a novel candidate oncogene and possibly a therapeutic target for several types of human cancer [5,7,12,16]. However, the abnormal expression of ATAD2 and its possible carcinogenesis in HCC have not been studied thus far. IHC showed that ATAD2 expression in the HCC tissues was significantly higher than in adjacent non-tumor tissues. In addition, the upregulation of ATAD2 was observed in the majority of HCCs compared to their adjacent normal liver tissues by Western blotting. These findings provide evidence that the upregulation of ATAD2 may play an important role in HCC tumorigenesis.

Further correlation analyses indicated that the high expression of ATAD2 in the HCC tissues was positively correlated with tumor metastasis. These results demonstrated that the upregulation of ATAD2 in HCC might play an important role in promoting malignant tumors. Similar results were also observed in other human malignancies, such as esophageal, gastric, colon, and breast cancers [12,16], in which the overexpression of the ATAD2 gene was often observed in more aggressive tumor subgroups and provided diagnostic value. Furthermore, we found that the high expression of ATAD2 in HCC was a strong and independent predictor of shortened overall survival.

With regard to the gene function, the bromodomain encoded by ATAD2 contributes to the high-affinity recognition of acetylated lysine [16], which is frequently found in chromatin remodeling and histone acetylation [17]. The bromodomain may play an important role in cell growth, proliferation, invasion and apoptosis. In the present study, ATAD2 protein was detectable by Western blotting in all seven cell types studied. The Huh7 and HCCLM3 cells had relatively higher levels of endogenous ATAD2, and the depletion of ATAD2 by transfection with siRNA in these two cell lines led to a G1 phase cell cycle arrest and reduced cell growth/proliferation. In addition, siRNA-mediated ATAD2 knockdown could significantly inhibit cell migration and cell invasion. This result was in agreement with previous studies that showed that ATAD2 was closely involved in several key regulatory mechanisms to control cell proliferation or tumor metastasis through its structure domain [18,19]. Taken together, these data provide evidence that ATAD2 is not only important in HCC cell proliferation but also involved in carcinoma cell migration and invasion.

However, the molecular mechanisms by which ATAD2 regulates cancer cell proliferation and invasion remain unclear. From the Human Tumor Metastasis RT-PCR array with ATAD2 siRNA-treated cells, we identified APC, ITGB3, FXYD5, ITGA7 and CTNNA1 (α-catenin), which are related to cell adhesion; among these, the expression of APC and CTNNA1 changed by at least 3-fold.

APC, which negatively regulates β-catenin, plays an important role in the development of HCC. In mice lacking APC in the liver, β-catenin was stabilized, which significantly increased the incidence of HCC [20]. Colnot et al. [20] found that 67% of APC(lox/lox) mice developed HCC when Cre adenovirus was injected. The β-catenin signaling in these mice was strongly activated, indicating that the loss of APC function led to Wnt signaling pathway activation and caused the occurrence of tumors.

CTNNA1 functions in a complex with CTNNB1, where it acts to tether the cytoplasmic domain of E-cadherin to the cytoskeleton [21,22]. Vasioukhin et al. found that inhibiting CTNNA1 destablizes adherens junctions, which weakens the interaction between cells and makes them less sensitive to contact-mediated inhibition [23]. Previous studies showed that reduced CTNNA1 expression in some solid tumors may be associated with increased malignant behavior [24-26]. However, some studies showed that CTNNA1 was more highly expressed in poorly differentiated HCC than in well-differentiated HCC [27,28].

CTNNA1 and CTNNB1 link cadherins to the cytoskeleton at epithelial cell-cell adherens junction complexes [29]. As is well known, in addition to the formation of the E-cadherin-catenin complex, CTNNB1 is associated with the Wnt/Wingless signal transduction pathway and binds to APC, EGFR and the c-erbB2 proto-oncogene. Thus, whether ATAD2 could affect APC and CTNNA1 to foster tumorigenesis by activating the WNT pathway is a goal for further research.

In the present study, we detected changes in the expression of two genes—upregulated APC and downregulated CTNNA1—after knocking down ATAD2 in Huh7 cells, and we validated these changes at the protein level by Western blotting. It appears that in our HCC cells, ATAD2 regulates cell migration/invasion via the regulation of APC and/or CTNNA1 expression. Through real time PCR detection in 45 paired tissue samples, we also found that ATAD2 was significantly negatively correlated with APC and CTNNA1 expression.

Moreover, we also studied the upstream mechanism regulating ATAD2 expression in HCC. miR-372 belongs to the miR-371-373 gene cluster that also includes miR-93 and miR-302a [30]. These microRNAs play an important role in the development of many types of human malignant tumors. One report noted that miR-372 is an oncogene in human glioma; it is more highly expressed in glioma cells than in normal brain cells and is closely related to the prognosis of glioma [31]. In the gastric cancer cells, Cho et al. found that miR-372 could promote cell proliferation and inhibit apoptosis; these biological roles are caused by acting on the downstream LATS2 gene [32]. LATS2, which is an important tumor suppressor, plays an important role in inhibiting cell proliferation in gastric cancers. In the present study, we found that miR-372 was negatively correlated with ATAD2. Through the luciferase reporter gene assay, we further confirmed that the ATAD2 mRNA 3′ non-coding region (3′ UTR) has a binding site for miR-372. More work is necessary to understand how miR-372 regulates ATAD2 expression.

## Conclusions

In summary, we provided a basis for the concept that the high expression of ATAD2 in HCC may be important in the acquisition of an aggressive phenotype and indicate a poor prognosis for HCC patients. Furthermore, the functional studies of ATAD2 in this report suggest a potential role of ATAD2 in affecting cell proliferation, invasion, and migration and make ATAD2 an attractive target for future cancer therapeutics.

## Competing interests

The authors declare that they have no competing interest.

## Authors’ contributions

HYL, HH, YYW, XJL, SGX and XXM carried out the experimental work, YQY provided data analysis and histopathological analysis, YFL provided tumor samples, clinical information, GW designed the study and participated in writing the paper. All authors read and approved the manuscript.

## Pre-publication history

The pre-publication history for this paper can be accessed here:

http://www.biomedcentral.com/1471-2407/14/107/prepub

## Supplementary Material

Additional file 1: Table S1 Genes differentially expressed in HCC after ATAD2 knockdown according to a Human Tumor Metastasis Real-time PCR Array. Click here for file
