# Supplementary material for: miR-372 down-regulates the oncogene ATAD2 to influence hepatocellular carcinoma proliferation and metastasis
Source: BMC Cancer. 2014 Feb 19;14:107. doi: 10.1186/1471-2407-14-107 (PMC4016509; doi:10.1186/1471-2407-14-107)
Supplement: Additional file 1: Table S1 — Genes differentially expressed in HCC after ATAD2 knockdown according to a Human Tumor Metastasis Real-time PCR Array. [file 1471-2407-14-107-S1.doc]

**Additional file 1: Table S1.** **List of genes differentially expressed in hepatocellular carcinoma HCCLM3 cells after ATAD2 knockdown using a Human Tumor Metastasis Real-time PCR Array**

| ***Gene*** | ***Fold change*** | ***Location*** | ***Function*** |
| --- | --- | --- | --- |
| Upregulated genes | |  |  |
| APC | 5.6569 | 5q21-q22 | cell adhesion, inhibits proliferation |
| ITGB3 | 2.969 | 17q21.32 | participates in cell adhesion |
| TNFSF10 | 2.4623 | 3q26 | induces apoptosis, inhibits proliferation |
| KISS1 | 2.4453 | 1q32 | suppresses metastasis |
| CTBP1 | 2.0562 | 4p16 | inhibits cell proliferation |
| FXYD5 | 2.0562 | 19q12-q13.1 | negative regulation of cell adhesion |
| MTA1 | 1.9588 | 14q32.3 | promotes metastasis |
| MMP2 | 1.9185 | 16q13-q21 | decomposes, protein hydrolysate and promotes metastasis |
| TIMP4 | 1.879 | 3p25 | inhibits metastasis |
| CXCL12 | 1.8277 | 10q11.1 | participates in cell adhesion |
| TCF20 | 1.7901 | 22q13.3 | transcription factor |
| CD82 | 1.7532 | 11p11.2 | metastasis suppressor |
| CXCR2 | 1.7532 | 2q21 | signal transduction, promotes invasion |
| MMP10 | 1.7532 | 11q22.3 | protein hydrolysate, promotes metastasis |
| CCL7 | 1.7053 | 16q13 | promotes invasion and migration |
| CDH11 | 1.7053 | 16q22.1 | involves in the metastatic process |
| CXCR4 | 1.7053 | 2q21 | promotes invasion and migration |
| FLT4 | 1.7053 | 5q34-q35 | promotes tumor metastasis |
| HGF | 1.7053 | 7q21.1 | participates in proteolysis, promotes proliferation |
| HPSE | 1.7053 | 4q21.3 | hydrolyses protein |
| IGF1 | 1.7053 | 12q22-q23 | promotes proliferation |
| KISS1R | 1.7053 | 19p13.3 | suppresses metastasis |
| MMP3 | 1.7053 | 11q22.3 | decomposes, protein hydrolysate and promotes metastasis |
| MMP9 | 1.7053 | 20q11.2-q13.1 | decomposes, protein hydrolysate and promotes metastasis |
| MYCL1 | 1.7053 | 1p34.2 | transcription factor, promotes proliferation |
| NR4A3 | 1.7053 | 9q22 | transcription factor, promotes proliferation |
| RORB | 1.7053 | 9q22 | participates in regulate of transcription |
| TSHR | 1.7053 | 14q31 | promotes proliferation |
| SERPINE1 | 1.5911 | 7q22.1 | promotes metastasis |
| FN1 | 1.5476 | 2q34 | participates in cell adhesion |
| TIMP2 | 1.5369 | 17q25 | inhibits metastasis |
| MDM2 | 1.5052 | 12q14.3-q15 | negative regulation of cell proliferation |
| CD44 | 1.4845 | 11p13 | cell adhesion and stroma attachment |
| CST7 | 1.4845 | 20p11.21 | inhibits cysteine proteinase |
| TRPM1 | 1.4845 | 15q13-q14 | calcium channels |
| GNRH1 | 1.4743 | 8p21-p11.2 | inhibits cell proliferation |
| HRAS | 1.4641 | 11p15.5 | promotes proliferation |
| CTSL1 | 1.3851 | 9q21-q22 | protein hydrolysate |
| NF2 | 1.3851 | 22q12.2 | inhibits proliferation |
| CHD4 | 1.3755 | 12p13 | chromatin assemble and modification |
| RPSA | 1.3472 | 3p22.2 | participates in cell adhesion |
| MMP11 | 1.3379 | 22q11.23 | decomposes, protein hydrolysate and promotes metastasis |
| PLAUR | 1.3104 | 19q13 | activator of plasminogen |
| MGAT5 | 1.2924 | 2q21 | promotes metastasis |
| CTSK | 1.2746 | 1q21 | protein hydrolysate |
| TGFB1 | 1.2483 | 19q13.1 | inhibits or promotes proliferation, promotes metastasis |
| FGFR4 | 1.2397 | 5q35.1 | promotes invasion |
| MCAM | 1.2397 | 11q23.3 | participates in cell adhesion |
| SRC | 1.2058 | 20q12-q13 | promotes proliferation |
| VEGFA | 1.1728 | 6p12 | promotes proliferation, metastasis; inhibits apoptosis |
| CDH1 | 1.1647 | 16q22.1 | inhibits tumor metastasis |
| BRMS1 | 1.1096 | 11q13-q13.2 | inhibits metastasis and tumor growth |
| MTSS1 | 1.1096 | 8p22 | inhibits metastasis and proliferation |
| MET | 1.0943 | 7q31 | Proto-oncogene, promotes cell proliferation |
| EPHB2 | 1.0644 | 1p36.1-p35 | signal transduction, promotes invasion |
| TIMP3 | 1.0644 | 22q12.3 | induces apoptosis, inhibits metastasis |
| SMAD2 | 1.057 | 18q21.1 | cell signal transduction |
| TP53 | 1.0497 | 17p13.1 | induces apoptosis and cell differentiation, inhibits proliferation |
| NME4 | 1.0353 | 16p13.3 | inhibits proliferation |
| IL1B | 1.021 | 2q14 | inhibits or promotes proliferation |
| Downregulated genes | | | |
| ETV4 | -1.007 | 17q21 | transcription factor, promotes proliferation |
| MMP7 | -1.007 | 11q21-q22 | decomposes, protein hydrolysate and promotes metastasis |
| SMAD4 | -1.007 | 18q21.1 | cell signal transduction |
| DENR | -1.0281 | 12q24.31 | promotes proliferation |
| GAPDH | -1.0562 | 12p13 | glycometabolism |
| IL18 | -1.0644 | 11q22.2-q22.3 | promotes cell proliferation |
| EWSR1 | -1.0792 | 22q12. | transcription factor, promotes oncogenesis |
| CDKN2A | -1.1173 | 9p21 | negative regulation of cell cycle |
| SYK | -1.1408 | 9q22 | promotes proliferation |
| PNN | -1.1728 | 14q21.1 | inhibits proliferation |
| COL4A2 | -1.2483 | 13q34 | the component of extracellular matrix |
| RB1 | -1.2746 | 13q14.2 | negative regulation of cell reproduction |
| FAT1 | -1.3013 | 4q35 | participates in cell adhesion |
| HPRT1 | -1.3195 | Xq26.1 | nucleotide metabolism |
| KRAS | -1.3379 | 12p12.1 | cell signal transduction, proliferation |
| MYC | -1.3851 | 8q24.12-24.13 | promotes proliferation |
| NME1 | -1.3851 | 17q21.3 | negatively regulates proliferation and participates in cell adhesion |
| ACTB | -1.5052 | 7p15-p12 | ORM cytoskeleton |
| PTEN | -1.5583 | 10q23.3 | inhibits proliferation and metastasis |
| HTATIP2 | -1.6021 | 11q13 | positively regulates transcription |
| CDH6 | -1.6702 | 5p15.1-p14 | osteosis, cell adhesion |
| B2M | -1.879 | 15q21-q22.2 | immune response, MHC I receptor |
| METAP2 | -1.9725 | 12q22 | protein hydrolysate and modification |
| SET | -2.395 | 9q34 | inhibits histone acetylation |
| ITGA7 | -2.5847 | 12q13 | participates in cell adhesion |
| SSTR2 | -3.0951 | 17q24 | inhibits proliferation |
| MMP13 | -3.4822 | 11q22.3 | decomposes, protein hydrolysate and promotes metastasis |
| CTNNA1 | -3.5801 | 5q31 | participates in cell adhesion |
